# Beach boys in Galle, Sri Lanka: multiple HIV risk behaviours and potential for HIV bridging

**DOI:** 10.1186/s12889-020-09699-x

**Published:** 2020-10-23

**Authors:** Ivana Bozicevic, Ariyaratne Manathunge, Sriyakanthi Beneragama, Chathrini Gadjaweera

**Affiliations:** 1 World Health Organization Collaborating Centre for HIV Strategic Information, University of Zagreb School of Medicine, Zagreb, Croatia; 2 National STD/AIDS Control Programme, Ministry of Health, Colombo, Sri Lanka

**Keywords:** HIV, Transactional sex, Tourists, Sri Lanka

## Abstract

**Background:**

There are limited data globally on HIV in men who engage in casual and transactional sex with female tourists.

**Methods:**

In 2018 we carried out a respondent-driven sampling (RDS) survey among beach boys in Galle, Sri Lanka, to determine prevalence of HIV and other infections, HIV risk behaviours and utilisation of HIV prevention services. Eligibility criteria included men who cruise in and around beach areas and who had anal and/or vaginal sex with female or male tourists in the 12 months before the survey.

**Results:**

We recruited 373 beach boys. Approximately 49.6% of the participants were married, while 45.7% were single and 4.7% divorced, separated or widowed. A lower percentage of beach boys reported regular partners in the past 12 months (52.3%) compared to casual partners (95.4%). Condom use at last sex with a casual partner was higher (76.7%) compared to condom use with regular partners (58.3%). Condom use at last sex with a tourist was reported by 75.3%. Ever receiving money, goods or services in exchange for sex was reported by 39.7%. For 85.5% of beach boys who sold sex, the last paying partner was a tourist (85.5%) and a woman (82.0%). In the past 12 months before the survey, 32.3% of beach boys paid money for sex, and 99.5% did so from women.

Ever been tested for HIV was reported by 35.3, and 69.1% of those were tested in the 6 months before the survey. In the adjusted multivariate analysis, significant correlates of never testing for HIV were lack of comprehensive knowledge about HIV and unprotected last sexual intercourse with tourists.

The prevalent infections were: HIV, 0.3% (95% CI 0.0–0.4%); syphilis, 0.5% (0.0–1.2%); herpes virus type-2, 5.0% (2.5–7.5%).

**Conclusions:**

There are low level HIV and syphilis prevalence among beach boys in Galle but a high level of sexual risk taking. Beach boys may be acting as a bridge for HIV transmission between higher-risk groups (paying female tourists, men who have sex with men) and lower-risk heterosexual female population in Sri Lanka. More research is needed in South-East Asia on men who trade sexual services to female and male tourists.

## Background

It was estimated that 3500 [3000–4100] adults aged 15 years and above were living with HIV in Sri Lanka at the end of 2018, and HIV prevalence among adults 15–49 years old was < 0.1% [1]. In 2018, 350 cases of HIV were reported [2]. In the period 2011–2018 the number of reported cases increased from 78 to 285 in men, respectively, while around 60 cases were annually reported in women.

National HIV Strategic Plan for Sri Lanka for 2018–2022 identifies female sex workers (FSW), men having sex with men (MSM), transgender (TG) persons, beach boys, people who use drugs and people who inject drugs (PWID) as key populations for HIV transmission [3].

From March – June 2018, we conducted the second round of an integrated bio-behavioural survey (IBBS) using respondent-driven sampling (RDS) among beach boys in Galle in order to determine the prevalence of HIV, syphilis and herpes virus type-2 (HSV-2), corresponding risk behaviours and utilization of HIV prevention services. Beach boys refer to men who work or socialise on the tourist beaches or close to the beaches, and who offer sex to men and women in exchange for money or some form of gratification. Many of them work in the tourism industry, in restaurants, hotels, guest houses and boat-related tourism.

Galle is a coastal city in south-western Sri Lanka with a rapidly growing tourism industry. In 2010 nearly 654,400 tourists visited Sri Lanka, increasing to 2,333,800 in 2018, which is over 350% growth in 9 years [4]. During 2018, out of the total 12,608,044 foreign tourist nights in graded accommodation establishments, 4,521,094 (35.9%) were in the South Coast resort region where the district of Galle is situated [5].

There is evidence suggesting that international tourists are more likely to engage in risk-related sexual behaviours when outside their own community [6, 7]. Vivancos et al. showed in the systematic review that casual sex while travelling is relatively common, although this varies by the country of residence, destination, and nature of travel [8]. In Great Britain, 9.2% of men and 5.3% of women reported new sexual partners while abroad in the preceding 5 years [9]. The research on sex tourism has expanded its focus from solely men to also exploring women as clients of men who sell sex and the implications this has on recipient communities [10–14]. The recent systematic mapping review on women who trade sexual services from men found that studies that involve tourist women as buyers of sex from men were done in only a few countries - Kenya, Jamica, the Dominican Republic, Gambia, Ghana, India, Indonesia, and Tunisia. Most of these studies were written from anthropological or sociological perspective with little focus on HIV and sexually transmitted infections (STIs).

In this paper, we report on the prevalence of HIV, syphilis and HSV-2 infection, risk behaviours and correlates of HIV testing among beach boys in Galle. We also provide some comparisons of HIV behavioural and service-utilisation indicators with the first IBBS in beach boys carried out in Galle in 2015 [15]. In 2015, 306 beach boys were recruited in an RDS-based IBBS [15].

## Methods

### Study setting and sampling

The study was carried out in Galle, Sri Lanka, on the premises of the non-governmental organization (NGO) Samadhi Foundation.

RDS was used as a sampling method. The criteria for inclusion in the survey included the following: men who cruise in and around beach areas and who had anal and/or vaginal sex with female or male tourists in the 12 months before the survey.

RDS is a chain-referral sampling method used worldwide to recruit ‘hidden’ populations that are less likely to be recruited from public venues. The theoretical and mathematical foundations and procedures of RDS have been well established in published literature [16, 17].

The recruitment in our survey began with three pre-selected, socially well networked participants (so called “seeds”). Seeds were given three coupons by study staff to enroll up to three other beach boys from their social networks. Subsequent participants also received up to three coupons and were told by study staff to give these coupons to their peers. Participants received a primary incentive of 2US$ for their participation in the survey and a secondary incentive of 1US$ for each person they recruited and who successfully completed the survey.

Recruitment continued until the sample size and equilibrium on key indicators were reached.

Participants provided verbal informed consent before participating and were screened for eligibility. After completing a questionnaire, participants received HIV pre-test counselling and afterwards provided biological specimens for testing for HIV and other infections. All participant information was anonymous and confidential. Three weeks after testing, participants could receive test results. All respondents whose test results were reactive were referred to the closest STI clinic for further evaluation, management and follow up, as needed.

Sample size calculation was set up to detect a 15% increase in consistent condom with non-regular partners during a year before the survey from a baseline level of 35.2%, with 80% power and an alpha error of 5%. This gave a sample size of 336 respondents.

### Behavioural questionnaire

We used the same behavioural questionnaire as in the 2015 survey with minor modifications**,** which were done according to the World Health Organization (WHO) - recommended questionnaires for bio-behavioural HIV surveys in key populations [18]. The questionnaire used in the IBBS carried out in 2015 was published by the Family Health International [19]. Indicators needed for the Global AIDS Monitoring (GAM) were collected based on the questionnaire [20].

The questionnaire was completed in a face-to-face interview using a tablet with Open Data Kit. The questionnaire included questions regarding socio-demographic characteristics, social network sizes, sexual and drug using behaviours, knowledge about HIV and HIV testing history. The size of sexual network was assessed as the number of beach boys that participants know by name, are older than 18 years, live in Galle and have seen them in the past 1 month.

Coverage with HIV interventions was defined according to the GAM guidelines, as receipt of at least two out of the following HIV prevention services from an NGO or a health care provider in the past 3 months: condoms and lubricants; counselling on condom use and safe sex; testing for STIs [20].

### Laboratory tests

HIV testing was done in line with the WHO and the national algorithm for HIV testing, using Alere Determine HIV (Alere Determine HIV-1/2 Ag/Ab Combo, Abbott Laboratories, USA) rapid test kit as the first, and in case of reactivity SD Bioline HIV 1/2 rapid test (SD Bioline HIV-1/2 3.0, Global Diagnostics, India) as the second, and ABON Tri-Line HIV Rapid Test (ABON Biopharm Hangzhou Co., China) as the third test.

Determine Syphilis TP rapid test (Alere Determine™ Syphilis TP, Abbott Laboratories, USA) was used as the first test for syphilis, and those with reactive test results were tested using Venereal Disease Research Laboratory (VDRL) test (titre > 8 was considered indicative of active syphilis). Herpes Simplex Virus Type-2 IgG ELISA test (IBL International GmBH, Germany) was used to test for HSV-2.

For quality assurance purposes, every 10th HIV and syphilis-negative sample was re-tested at the National Reference Laboratory of the National STI and AIDS Control Programme (NSACP).

### Data analysis

The sample and estimated population proportions with 95% confidence intervals (CI) were calculated using RDS Analyst (RDS-A) software version 0.61 [21].We used the Gile’s sequential sampler (Gile’s SS) estimator for the analysis, which takes into account self-reported network sizes, recruitment patterns and estimated population size [22].

Homophily, convergence/equilibrium and bottlenecks were analyzed for key indicators (knowing HIV status from an HIV test, condom use at last sex with a casual partner, coverage with HIV prevention programs, discriminatory attitudes towards PLHIV and age).

Stata’s 16.1 was used for unweighted bivariate and multivariate logistic regression analysis due to concerns that weighted regression of RDS data does not perform well [23]. Logistic regression was employed to determine correlates of never testing for HIV. Independent variables used in the model included socio-demographic factors, knowledge about HIV and indicators of high-risk sexual practices. Knowledge about HIV prevention was assessed according to the guidelines for reporting of the GAM indicators. HIV knowledge indicator was constructed from responses to a set of five questions about ways of HIV transmission: „Score 5″ was given to those who answered correctly to all five questions, while “score 0–4” was given to those with at least one incorrect answer.

In the final logistic regression model we included variables associated with the outcome at *p* < 0.05 in the bivariate analysis as well as age. The cut-off for considering a result to be statistically significant in the multivariate analysis was set up at *p* = 0.05.

Chi-square test was done to determine whether there was a significant difference in values of behavioural and prevention indicators measured in 2018 compared to 2015.

### Ethical considerations

The protocol for the IBBS was submitted for ethical approval to the Ethical Committee of the Medical Faculty of the University of Sri Jayewardenepura, and the approval was obtained in October 2017. The Ethical Committee approved that respondents provide verbal consent for participation in the survey in order to avoid identification of individuals had a written consent been provided.

## Results

### Recruitment patterns

A total of 373 beach boy respondents were recruited in Galle from February–May 2018, including three seeds. There were eight waves of recruitment. The homophily on key variables ranged from 0.80 to 1.54 and convergence was reached on all the five variables examined.

The median network size (the number of beach boys older than 18 years who live in Galle that participants knew and met in the past 1 month) was 13 (range: 2–50).

### Socio-demographic characteristics

The median age of beach boys in Galle was 30.0 years (range: 18–72). In regular work were 61.8% of respondents, while occasional work and being unemployed were reported by 31.5 and 6.2%, respectively. Almost a half of participants were married (49.6%), while 45.7% were single, and 4.7% divorced, separated or widowed. All respondents were born in Sri Lanka and Galle was a district of residence in the year before the survey for the vast majority. Never attending school was reported by 2.4%, a completed primary school by 49.6% and a completed secondary school education by 48.0% of participants.

### Sexual and drug using behaviours

A lower percentage of beach boys reported regular partners in the past 12 months (52.3%) compared to casual partners (95.4%) (Table 1). Condom use at last sex with a casual partner was higher (76.7%) compared to condom use with regular partners (58.3%). In the 7 days before the survey, beach boys reported a median of two sexual partners (range: 0–20), while in the 12 months preceding the survey they had a median of 6.0 partners (range: 1–50).

**Table 1:** Comparison of indicators related to sexual behaviours and testing for HIV in beach boys in Galle; Integrated HIV bio-behavioural surveys carried out in 2015 and 2018

	2015		2018		X^2^	*p*-value
	n/ N	% (95% CI)^a^	n/ N	% (95% CI)		
Used condom at last sex with a casual partner	211/292	67.1 (60.2–72.8)	264/364	76.7 (72.8–80.7)	0.01	0.939
Last casual sexual partner’s HIV status unknown	179/292	60.7 (54.9–66.5)	249/366	70.6 (66.0–75.2)	3.24	0.071
Used condom at last sex with a regular partner	130/ 206	61.2 (53.2–68.3)	124/223	58.3 (51.3–65.8)	2.50	0.114
Last regular partner’s HIV status unknown	Not available		48/224	22.1 (17.4–26.9)		
Used condom at last sex with a tourist	212/298	67.6 (61.8–73.3)	259/362	75.3 (71.3–79.4)	0.01	0.909
Used condom at last sex for which money was received	49/97	49.3 (39.1–59.1)	120/158	77.0 (70.1–83.9)	17.40	< 0.001
Used condom at last sex for which money was given	44/71	60.1 (46.7–72.8)	101/142	70.7 (60.3–80.7)	1.83	0.176
Was paid for sex at last sex with a tourist	82/290	25.6 (20.7–30.3)	93/366	24.4 (20.2–28.5)	0.67	0.410
Ever tested for HIV	28/306	10.4 (6.7–14.5)	117/336	35.3 (29.3–41.3)	60.42	*p* < 0.001
Discussed HIV with any sexual partner (among those who heard of HIV)						
Yes, all	8/211	3.3 (1.1–5.4)	2/249	0.4 (0–7.4)	54.52	< 0.001
Yes, some	58/211	25.2 (19.4–30.2)	13/249	4.7 (3.0–7.1)		
No, none	141/211	69.3 (63.7–75.6)	215/249	85.6 (81.3–89.6)		
Don’t know	4/211	2.3 (0.2–4.5)	19/249	9.2 (5.7–13.1)		

Totals vary because of missing data or subgroup analysis

X^2^ = chi-square test

^a^Weighted population estimates and confidence intervals (CI)

Ever having anal sex with another man was reported by 16.0% and ever injecting drugs by 2.9%.

For one in four beach boys last sexual intercourse with a tourist was unprotected, and the commonest explanations given for this were non-availability of condoms (51.9%) and thinking that condom use was not necessary (42.1%).

In terms of sexual relationships with tourists, analysis by marital status revealed that among married beach boys (*n* = 152), 83.7% have only female tourists as partners, 6.0% only male tourists and 10.2% both male and female tourists. Among never married and single (*n* = 156), 75.7% reported only female tourists as partners, 7.9% only male and 16.4% both male and female tourists. Those in the other marital categories (divorced, widowed and separated) reported only female tourists as partners.

About one-third (39.7%) of beach boys ever received money, goods or services in exchange for sex, and the majority (94.7%) did so in the 12 months before the survey. For a majority of beach boys in Galle that sold sex, the last paying partner was a tourist (85.5%) and a woman (82.0%). Tourists that beach boys had sex with in 12 months before the survey were most frequently from Germany, Russia, France and Thailand. Amount of money typically received in exchange for sex was 21 USD.

In the past 12 months before the survey, 32.3% of beach boys paid money for sex, and the majority (99.5%) did so from women.

Compared to the first IBBS carried out in 2015, some HIV-related behavioural and prevention indicators significantly improved in 2018, such as condom use at last sex for which money was received and testing for HIV (Table 1). In contrast to these improvements, the proportion of beach boys who reported not knowing HIV status of last casual partner increased when 2018 is compared to 2015.

### Coverage with prevention services, including HIV testing

Approximately one in three respondents (38.4%) correctly identified both ways of preventing the sexual transmission of HIV and rejected major misconceptions about HIV transmission.

Somewhat more than a half of participants (58.3%) do not consider themselves to be at risk of HIV while 31.4% said that they could not estimate whether or not they were at risk. Only 7.3% estimate their risk to be moderate or high.

Ever been tested for HIV was reported by 35.3, and 69.1% of those were tested in the 6 months before the survey. Of those tested, 94.5% indicated a negative test results while the others reported not getting a test result. The majority of respondents (94.0%) were last tested for HIV at a governmental STI clinic. Between 2015 and 2018, uptake of HIV testing among beach boys substantially increased (Table 1).

The commonest reasons given for not testing for HIV was lack of time (45.0%) and not being at risk of HIV (32.9%), followed by an inconvenient testing location (16.0%).

Overall, coverage with HIV prevention, as defined by GAM, among beach boys was 14.7%.

Table 2 shows correlates of ever being tested for HIV using bivariate and multivariate regression analysis. After adjustment in the multivariate analysis, significantly lower odds of never testing for HIV were found among beach boys who demonstrated the most comprehensive knowledge about HIV (aOR = 0.19; 95% CI 0.11–0.33) compared to those with at least one incorrect answer on knowledge questions, and higher odds among those who did not use condom at last sexual intercourse with tourists for which they were paid compared to those who did use (aOR =4.33; 95% CI 1.76–10.68). Marginally lower odds of never testing for HIV were found among those with more than two partners in the past 12 months.

**Table 2:** Bivariate and multivariate logistic regression analysis of correlates of never being tested for HIV among beach boys in Galle, 2018

	n/N	OR (95% CI)	***P*** value	aOR (95% CI)	***P*** value
Age					
18–24	64/102	1		1	
25–40	92/157	0.84 (0.50–1.40)	0.505	0.83 (0.45–1.54)	0.561
≥ 41	63/77	2.67 (1.32–5.41)	0.006	0.98 (0.39–2.43)	0.965
Employment status					
In paid work	98/177	1		1	
Occasional or no work	110/146	2.46 (1.53–3.98)	< 0.001	1.44 (0.79–2.59)	0.229
Partnership status					
Living together with a partner/spouse	95/140	1.0		–	
In a stable relationship but not living together	26/46	0.62 (0.31–1.21)	0.164		
Not in a stable relationship	98/150	0.89 (0.55–1.46)	0.649		
Knowledge of HIV^a^					
Score 0–4	175/217	1		1	
Score 5 (All answers correct)	38/113	0.12 (0.07–0.20)	< 0.001	0.19 (0.11–0.33)	< 0.001
Number of partners in the past 12 months					
1–2	51/55	1		1	
3–4	43/61	0.19 (0.06–0.60)	0.005	0.28 (0.07–1.07)	0.06
> 5	125/220	1.10 (0.04–0.29)	< 0.001	0.31 (0.09–1.06)	0.0
Used condom at last sex with a tourist					
Yes	115/222	1		1	
No	95/103	11.05 (5.13–23.82)	< 0.001	4.33 (1.76–10.68)	< 0.001
Paid at last sex with a tourist					
No	168/247	1			
Yes	47/82	0.63 (0.38–1.05)	0.08	–	
Gender of tourists that beach boys have sex with					
Only women	195/294	1			
Both men and women, or only men	20/37	0.60 (0.30–1.19)	0.14	–	

*OR* odds ratio, *aOR* adjusted odds ratio, *CI* confidence intervals

^a^HIV knowledge was assessed according to the guidelines for Global AIDS Monitoring for the year 2018. This indicator is constructed from responses to a set of five questions: „Score 5″ was given to those who answered correctly to all five questions, while “score 0–4” was given to those with at least one incorrect answe

### Prevalence of HIV, syphilis and HSV-2

One person was found to be positive for HIV, giving an HIV prevalence of 0.3% (95% CI 0.0–0.4%). Treponemal antibodies were detected in 0.5% (0.0–1.2%) while only one person had a weakly reactive VDRL test results suggestive of an active syphilis infection. HSV-2 antibodies were found in 5.0% (2.5–7.5%).

## Discussion

Findings from this study demonstrate still low level of HIV and syphilis prevalence among beach boys in Galle but high level of sexual risk taking in a variety of contexts and relationships. HSV-2 prevalence was also relatively low, and lower compared to the estimate of 8% for the general population of South-East Asia [24].

HSV-2 epidemiology is the most relevant of all STIs to HIV epidemiology and since HSV-2 serology is a powerful marker of sexual risk behavior it should be a standard component of HIV surveillance surveys in such low-level HIV epidemic setting [25, 26].

An important finding from our study is that beach boys may be acting as a bridge for HIV transmission between higher-risk groups (paying female tourists, men who have sex with men) and lower-risk heterosexual female population in Sri Lanka. This is supported by the finding that up to 16% of married beach boys have sex with both male and female tourists. Some of these beach boys might be behaviourally bisexual due to stigmatisation of homosexuality and social norms and expectations to marry, as found in many Asian countries [27]. Some may be primarily attracted to women but have situational sex with men due to financial gain or experimentation [28–30].

A third of beach boys reported selling sex to female tourists in a year before the survey and one in four did not use a condom at last transactional sex with tourists. Sexual relationships between local men and tourist women are typically relationships between individuals who are unequal in terms of economic power. Complexity of these power relations and different expectations that partners in these relationships may have may lead to lower condom use [14]. Evidence suggests that some women holidaying in developing countries may have expectations of longer-term romantic relationships while some travel explicitly for sex [31, 32]. The existing studies on women sex tourists usually describe them as middle-class, well educated, while the local men as young, poorly educated, and from low socioeconomic backgrounds, typically working in the informal beach economy, exchanging sex part-time [33–35]. The majority of beach boys in our study were in their early 30-ies and did not have a regular employment.

In comparison with the IBBS done in 2015, there are encouraging findings of an increase in condom use with casual partners, tourists and during transactional sex, and increase in testing for HIV. However, ever been tested for HIV was reported by 35.3% of beach boys in 2018, which indicates sub-optimal testing coverage. Additionally, a smaller percentage of beach boys raised a topic of HIV with any of their sexual partners in the 2018 compared to the 2015 sample.

Multivariate analysis highlighted the importance that adequate knowledge of HIV has on HIV testing. Our findings complement those from other studies, which have found low HIV knowledge and risk perception to be a barrier to HIV testing [36–38]. Of note is that those who did not use a condom at last sex with tourists were less likely to tested for HIV, which may reflect lack of awareness of condoms as a means of HIV protection or low perception of risk of HIV.

### Need to expand HIV testing services

In such setting where HIV testing is sub-optimal, health care providers should take every opportunity to reinforce information about HIV transmission and prevention, and the benefits of early HIV diagnosis and early antiretroviral therapy initiation. That might empower beach boys to make more appropriate decisions about HIV testing. Recent innovations in testing approaches and technologies, such as availability of HIV self -testing, may also help to break down barriers to testing, such as inconvenience and concern about confidentiality, encouraging more regular testing [39]. Testing in communities where beach boys reside can be done during outreach activities at hotspots in mobile vans, during sporting and entertainment events [40]. Workplace testing might also be an effective strategy to reach employed beach boys and offer them HIV testing and other prevention services [41]. As the majority are in some form of employment, they can be provided with HIV interventions through an occupational health approach.

### Importance of increasing condom availability

Non-availability of condoms was mentioned by respondents as the main reason for not using condoms during transactional sex. This can be addressed by providing condoms through local health service delivery points, tea shops/hotels and general stores in conjunction with the implementation of awareness-raising activities. It is of paramount importance that targeted and effective interventions based on the combination of behavioral, biomedical, and structural approaches are kept sustainable and continue to be scaled up.

Due to increased tourism to South-East Asian countries, there is a need for larger scale studies that would enable more comprehensive understanding of female tourists’ sexual behaviours and the role that their sexual interactions with host communities has on HIV transmission From the perspective of HIV epidemiology, population groups such as beach boys and other groups of men who trade sex to women remain unexplored in the region.

### Study limitations

This study was not without limitation. Self-reported sexual behaviors are subject to social desirability and recall biases, and such biases also potentially limit comparisons of IBBS carried out in 2015 and 2018. RDS may have resulted in under-recruitment of participants with smaller social network sizes. As RDS is a quasi-representative sampling method, it is challenging to estimate to what extent study participants are truly representative of a population of beach boys. Non-random selection of seeds in RDS could lead to biased estimates. However, there were no problematic homophily values for the selected variables and the use of bootstrapped standard errors enabled to adjust for seed dependence in sampling process.

## Conclusion

The current evidence shows that the HIV epidemic in beach boys in Galle, Sri Lanka is still at low level, but there are multiple and overlapping risks for HIV transmission. This study contributes to a scant but growing body of evidence on HIV-related risk behaviours associated with female tourists. Increasing travel to Sri Lanka from countries and population groups with higher prevalence of HIV and other STIs, coupled with contexts which may amplify risks, creates an emerging priority for HIV interventions.

## Data Availability

The datasets used and/or analysed during the current study are available from the corresponding author on reasonable request.

## Abbreviations

aOR: adjusted odds ratio
CI: Confidence interval
FSW: Female sex worker
GAM: Global AIDS Monitoring
HSV-2: Herpes virus type-2
IBBS: Integrated bio-behavioural survey
MSM: Men who have sex with men
NGO: Non-governmental organisation
NSACP: National STI and AIDS Control Programme
PWID: People who inject drugs
RDS: Respondent-driven sampling
RDS-A: Respondent-driven sampling - Analysis tool
SS: Sequential sampler
STIs: Sexually transmitted infections
TG: Transgender
WHO: World Health Organization
